# Divergent prion strain evolution driven by PrP^C^ expression level in transgenic mice

**DOI:** 10.1038/ncomms14170

**Published:** 2017-01-23

**Authors:** Annick Le Dur, Thanh Lan Laï, Marie-George Stinnakre, Aude Laisné, Nathalie Chenais, Sabine Rakotobe, Bruno Passet, Fabienne Reine, Solange Soulier, Laetitia Herzog, Gaëlle Tilly, Human Rézaei, Vincent Béringue, Jean-Luc Vilotte, Hubert Laude

**Affiliations:** 1 Virologie Immunologie Moléculaires (VIM), INRA, Université Paris-Saclay, 78350 Jouy-en-Josas, France; 2 Génétique Animale et Biologie Intégrative (GABI), INRA, AgroParisTech, Université Paris-Saclay, 78350 Jouy-en-Josas, France

## Abstract

Prions induce a fatal neurodegenerative disease in infected host brain based on the refolding and aggregation of the host-encoded prion protein PrP^C^ into PrP^Sc^. Structurally distinct PrP^Sc^ conformers can give rise to multiple prion strains. Constrained interactions between PrP^C^ and different PrP^Sc^ strains can in turn lead to certain PrP^Sc^ (sub)populations being selected for cross-species transmission, or even produce mutation-like events. By contrast, prion strains are generally conserved when transmitted within the same species, or to transgenic mice expressing homologous PrP^C^. Here, we compare the strain properties of a representative sheep scrapie isolate transmitted to a panel of transgenic mouse lines expressing varying levels of homologous PrP^C^. While breeding true in mice expressing PrP^C^ at near physiological levels, scrapie prions evolve consistently towards different strain components in mice beyond a certain threshold of PrP^C^ overexpression. Our results support the view that PrP^C^ gene dosage can influence prion evolution on homotypic transmission.

## Abstract (web summary)

PrP^C^ protein plays a key role in prion transmission across species. Here, the authors compare transmission of a representative scrapie isolate to transgenic mice expressing variable levels of the same *Prnp* allele as the donor sheep, and find divergent strain propagation regulated by PrP^C^ gene dosage.

Mammalian prions are proteinaceous transmissible pathogens responsible for a broad range of lethal neurodegenerative diseases in animals and humans^1^, including scrapie in sheep and goats, bovine spongiform encephalopathy (BSE), cervid chronic wasting disease and human Creutzfeldt–Jakob disease (CJD). Prions are primarily formed of macromolecular assemblies of PrP^Sc^, a beta-sheet enriched conformer of the ubiquitously expressed, host-encoded prion protein PrP^C^ (ref. 2). During replication, PrP^Sc^ is thought to recruit and convert PrP^C^ molecules through a seeded-polymerization process^3^,^4^, leading to PrP^Sc^ aggregates deposition in the brain and sometimes in extraneural tissues and bodily fluids of the infected individuals^5^,^6^,^7^,^8^.

Multiple strains of prions can be recognized in a given host species, causing disease with specific phenotypic traits, such as time course to disease, neuropathological features, PrP^Sc^ biochemical properties and tissue or cell tropism^9^,^10^,^11^,^12^. There is compelling evidence that prion strain diversity reflects differences in PrP^Sc^ conformations, at the level of the tertiary and/or quaternary structure^4^. Although a dominant PrP^Sc^ conformation appears to determine the strain phenotype, there is evidence that differing, PrP^Sc^ three-dimensional conformations,—beyond the intrinsic diversity of PrP^Sc^ physicochemical states^13^,^14^,^15^,^16^—, may be co- propagated. This could reflect the presence of subdominant prion strains^17^,^18^,^19^,^20^ or a ‘quasi-species' phenomenon^11^,^21^, as documented for viral pathogens. Interspecies transmission of prions is usually limited by a species or transmission barrier^11^. However, such cross-species transmission events can occur, as exemplified by the emergence of variant CJD in the human population dietary exposed to BSE prions^1^. Based mostly on PrP transgenic mouse models data^22^, the conformational selection model^11^,^23^ posits that the transmission barrier stringency depends on the degree of steric compatibility between PrP^Sc^ structure(s) present in the infecting prion and host PrP^C^ conformational landscape. A high transmission barrier can lead to mutation-like events, generating prion variant(s) with new biological strain properties^24^,^25^,^26^,^27^,^28^. Oppositely, homotypic transmissions (that is, when the host expresses the same PrP amino acid sequence as the infecting prion) generally result in apparent lack of transmission barrier and conservation of strain type^24^,^29^,^30^,^31^. However, there are few, remarkable examples of abrupt, phenotypic change during homotypic prion transmission: a faster replicating strain was shown to emerge from serial propagation of laboratory mouse 87A prions on high dose-inoculated, wild-type mice^32^. More recently, experimental transmission of variant CJD cases to mice transgenic for human PrP occasionally produced a sporadic-like phenotype^31^. Finally, transmission of H-type atypical BSE prions to bovine PrP transgenic led to the emergence of prions with different strain phenotypes^33^,^34^.

Here, we examined the outcome of prion homotypic transmissions by comparing the transmission pattern of sheep prion isolates on multiple lines of transgenic mice expressing varying levels of the VRQ allele of ovine PrP, associated with the highest susceptibility to scrapie^35^. We show that either faithful or divergent prion strain propagation occurred, this being critically controlled by PrP^C^ gene dosage in the transgenic mouse brain. Our findings reveal a new facet of the biology of prions, with both practical and theoretical implications.

## Results

### Discordant strain phenotypes on PrP^C^ overexpresser mice

**Figure 1:**
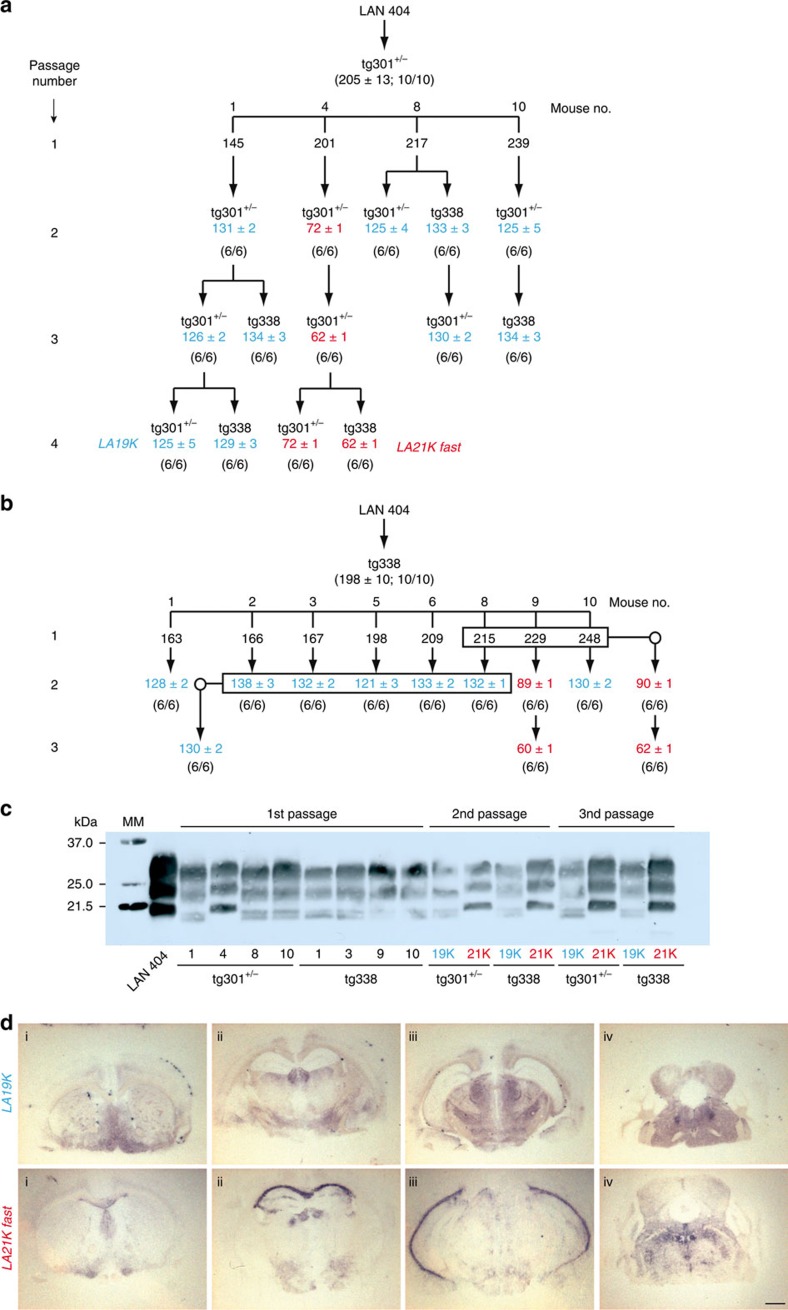
Serial transmission of LAN404 isolate to transgenic mice overexpressing ovine PrP. (**a**) Serial transmission of LAN404 primary, sheep scrapie isolate to *tg301*^+/−^ mice and (**b**) to *tg338* mice. Results of a representative experiment are shown. Blue and red colours are used to indicate the segregation of *LA19K* and *LA21K fast* phenotypes, respectively. (**c**) PrP^res^ electrophoretic patterns in the donor sheep and in *tg338* and *tg301*^+/−^ mouse brains following primary infection with LAN404 scrapie isolate and subsequent passage. (**d**) PrP^res^ deposition pattern in the brain of *tg338* mice infected with *LA19K* or *LA21K fast* prions. Representative histoblots of antero-posterior coronal brain sections at the level of the septum (i), hippocampus (ii), midbrain (iii) and brainstem (iv). In *LA21K fast*-infected mice, PrP^res^ deposited specifically in the septum, in the corpus callosum, and in certain raphe nuclei of the brainstem. In *LA19K*-infected mice, PrP^res^ deposits were finer and specifically detected in the caudate putamen, dorsal and posterior thalamic nuclei. Scale bar, 1 mm.

We transmitted by intracerebral route a hundred sheep isolates, with diverse geographical origin and proteinase K-resistant PrP^Sc^ (PrP^res^) signature, to *tg301*^+/−^ and *tg338* mice expressing VRQ ovine PrP^C^ at a level ∼8-fold that in sheep brain. *tg301*^+/−^ and *tg338* are independent lines expressing the same construct *tg3* at the hetero- and homozygous state, respectively^36^. *tg3* consists of a 125 kb piece of sheep DNA including regulatory sequences insert into a BAC vector, leading to a transgene expression pattern mimicking that of the endogenous gene^37^. Many isolates behaved as commonly observed when donor and host PrP sequences are homologous; that is, the incubation durations (ID) did not differ, or only showed moderate variations, between primary and subsequent passages. The PrP^res^ molecular profile in the brain was uniform among the inoculated mice, regardless of whether the donor sheep was of VRQ/VRQ genotype or not^9^,^30^,^36^. However, about half of the isolates from various genotypes, including several isolates sourced from an INRA experimental sheep flock where scrapie was highly prevalent (Langlade^35^), did not conform to this pattern. The data gathered in Fig. 1, obtained with the LAN404 isolate, illustrate the complex transmission pattern that can be seen with such isolates. On primary transmission, the ID averaged 200 days on both mouse lines, and was relatively homogenous, that is, 100% attack rate with SEM averaging 10 days (>10 independent inoculation experiments involving either *tg301*^+/−^ or *tg338* mice; Fig. 1a,b) Systematic examination for PrP^res^ content of the brains from terminally diseased *tg301*^+/−^ animals revealed unexpected molecular profiles. Nearly all mice exhibited a PrP^res^ signature clearly distinct from that in the donor sheep brain, with unglycosylated fragments migrating around 19 kDa (PrP^res^ 19K). A few mice—typically 1 out of 10—accumulated aglycosyl PrP^res^ sizing ∼21K (PrP^res^ 21K), as in the isolate (Fig. 1a–c). Secondary transmissions using individual *tg301*^+/−^ brains to the *tg301*^+/−^ or *tg338* lines consistently led to a marked shortening of the mean ID. Within at most two subpassages, the agent appeared to segregate under two readily distinguishable phenotypes, which we designated *LA19K* (mean ID ∼125–130 days and PrP^res^ 19K type) and *LA21K fast* (mean ID∼60 days and PrP^res^ 21K type). Serial transmission of LAN404 to *tg338* mice led to similar findings. In the experiment presented in Fig. 1b, all diseased mice showed a 19K PrP^res^ signature in the brain. Yet individual brain-to-brain subpassaging revealed that for one of them (no. 9) the PrP^res^ signature had shifted from 19K (Fig. 1c) to 21K type, and the mean ID shortened to ∼60 days within two subpassages. By contrast the ID stayed ∼130 days in the other passage series. This suggested that, in this mouse, one agent had outcompeted the other due to a much faster replication. Indeed, when subpassaging was performed using homogenates prepared from several brains, an agent with either *LA21K fast* or *LA19K* phenotype was propagated depending on whether mouse no. 9 was included in the pool or not (Fig. 1b). Thus, subpassaging LAN404 using brain pools from inoculated mice would have produced truncated information.

### Strain divergence is not due to a PrP^C^ sequence mismatch

The *LA21K fast* and *LA19K* strain phenotypes turned out to be fairly stable. Both strains could be propagated on *tg338* mice with no further change for up to 7 brain-to-brain subpassages, without the need of biological cloning by endpoint dilution. The clinical course was aggressive in *LA21K fast*-infected mice, in which hyperexcitability, hyperaesthesy, waddling and rolling gait dominated. In marked contrast, lethargy and hindlimb paresis dominated in *LA19K*-infected mice and the clinical course evolved at a slower pace. *LA21K fast* and *LA19K* strain phenotypes also differed with regard to PrP^res^ regional distribution within the brain, as assessed by histoblot on adjacent coronal sections (Fig. 1d). On the whole, our results were intriguingly reminiscent of the well-documented observation of dual strain evolution on cross-species transmission of transmissible mink encephalopathy to hamster. Such transmission leads to the emergence of two distinct, stable strains, hyper and drowsy, the phenotypic traits of which are similar to *LA21K fast* and *LA19K*, respectively^19^,^38^,^39^. We thus rechecked the transgene sequence to exclude the possibility that the isolation of *LA21K fast* and *LA19K* prions from LAN404 isolate transmission to transgenic mice resulted from a mismatch between host and donor PrP coding sequences. No difference was found between mouse and donor sheep DNAs, implying that the transmission was definitely homotypic. Therefore, the question arose as to what kind of selection pressures were enabling the sheep scrapie agent to adapt to its new, yet PrP homologous host.

### A dual cause for strain divergence in overexpresser mice

**Figure 2:**
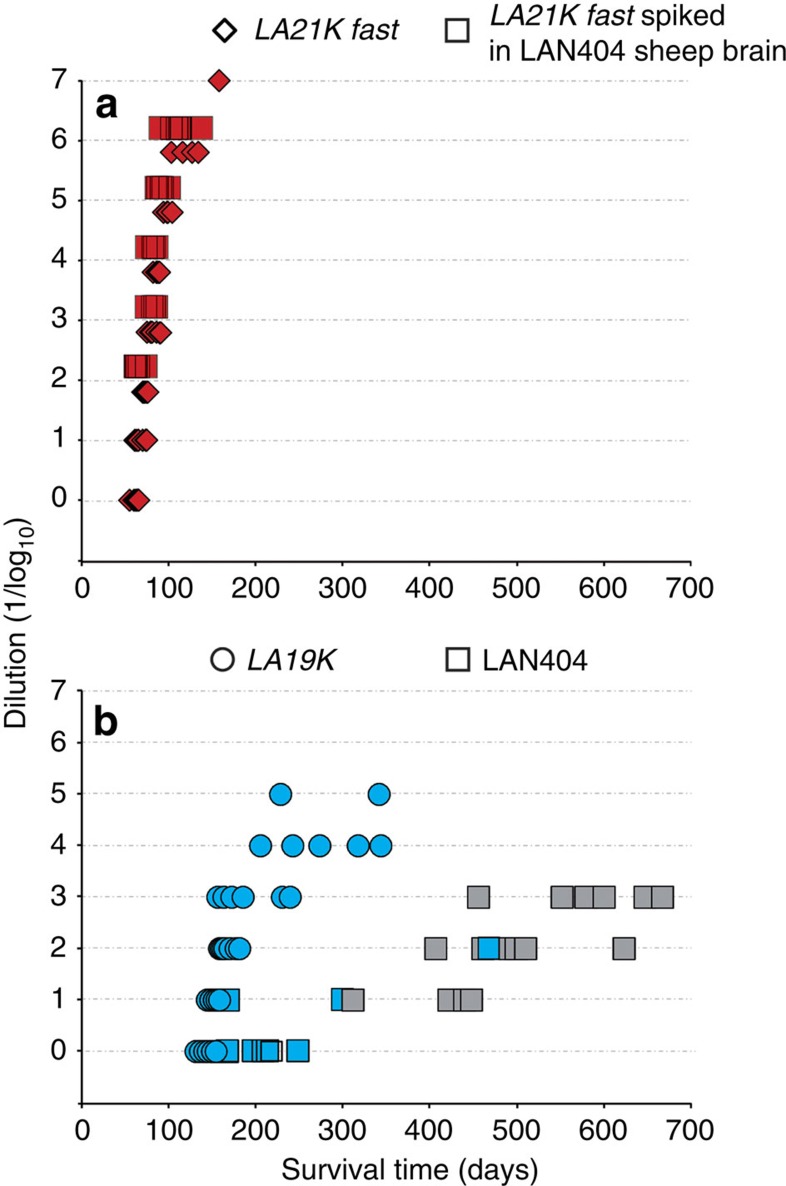
Infection dose/survival curve and PrP^res^ pattern in *tg338* mice infected with serially diluted homogenates. (**a**) Inoculation with serial tenfold dilutions of *tg338*-passaged *LA21K fast* brain material spiked (squares) or not (diamonds) in LAN404 sheep brain homogenate. The resulting curves are superimposable. (**b**) Inoculation with serial tenfold dilutions of LAN404 sheep (square) or *tg338*-passaged *LA19K* brain material (circles). Blue and red colours indicate presence of *19K* and 21K *fast* prions, respectively. Grey colour indicates a 21K PrP^res^ profile. The number of mice accumulating 19K and 21K PrP^res^ decreases and increases with dilution, respectively. (*n*=6 mice were inoculated per dilution).

We asked whether the emergence of the *LA21K fast* and *LA19K* strain components occurred during the propagation in the transgenic mice and/or reflected a pre-existing diversity in the sheep scrapie isolate. To investigate whether *LA21K fast* prions could pre-exist in LAN404 isolate, we challenged intracerebrally *tg338* mice with LAN404 brain homogenate spiked with serial 10-fold dilutions of *LA21K fast* prions (Fig. 2a). The result was a dramatic shortening of the mean ID, even at endpoint infectious titres of *LA21K fast* (∼120 instead of ∼200 days for unspiked homogenate (Fig. 1b). As a consequence, *LA21K fast* prions are unlikely to pre-exist in the sheep scrapie isolate (compare Fig. 1a,b).

We next reasoned that, should LAN404 isolate be populated by more than one strain component, diluting the inoculum might be a means to eliminate one of them. Figure 2b shows the individual ID and brain PrP^res^ signatures observed on *tg338* mice inoculated with serial dilutions of LAN404 sheep brain homogenate. Remarkably, the more diluted the inoculum, the higher the proportion of individuals with a prominent 21K-type signature, up to 100% (6/6) at the 10^−3^ dilution. Comparing this dose/ID curve with that obtained with stabilized *LA19K* prions (three passages on *tg338* mice, Fig. 2b) allowed us to calculate that the agent predominant in the original LAN404 isolate had a doubling time about 3-fold higher than *LA19K* prions. Altogether these data supported the view that *LA19K* agent is not created *de novo* on propagation in the overexpresser mice but instead pre-exists in the donor sheep isolate as a minor component, the replication of which is strongly favored in such mice. We also performed a subpassage from individual mice inoculated with 10^3^-diluted LAN404 brain material, culled at terminal stage of disease (>500 days post-inoculation, that is more than 400 days than ID of *LA21K fast* prions at endpoint dilution), all showing a 21K PrP^res^ signature in their brains (Fig. 2b). As illustrated in Supplementary Fig. 1, the outcome in *tg338* reporter mice was found to depend on the donor brain: either a long ID (∼130 days) with 21K PrP^res^ in most mice and 19K PrP^res^ in some mice, or a short ID (∼75 days) with 21K PrP^res^ in all mice. In addition, the 21K PrP^res^ molecular profiles as well as the deposition pattern in the brain noticeably differed among these two groups of mice. These results support the view that LAN404 sheep scrapie agent can replicate in high expresser mice, and that *LA21K fast* is a mutant emerging in a stochastic manner within one or two passages.

To summarize, the dual strain evolution observed on both overexpresser lines appears to involve two well distinct mechanisms: (i) preferential selection of an agent present in the donor sheep brain as a subdominant component; (ii) stochastic emergence of a variant that ineluctably outcompetes every other component due to its much greater replication efficiency.

### PrP^C^ gene dosage determines the divergent strain evolution

**Figure 3:**
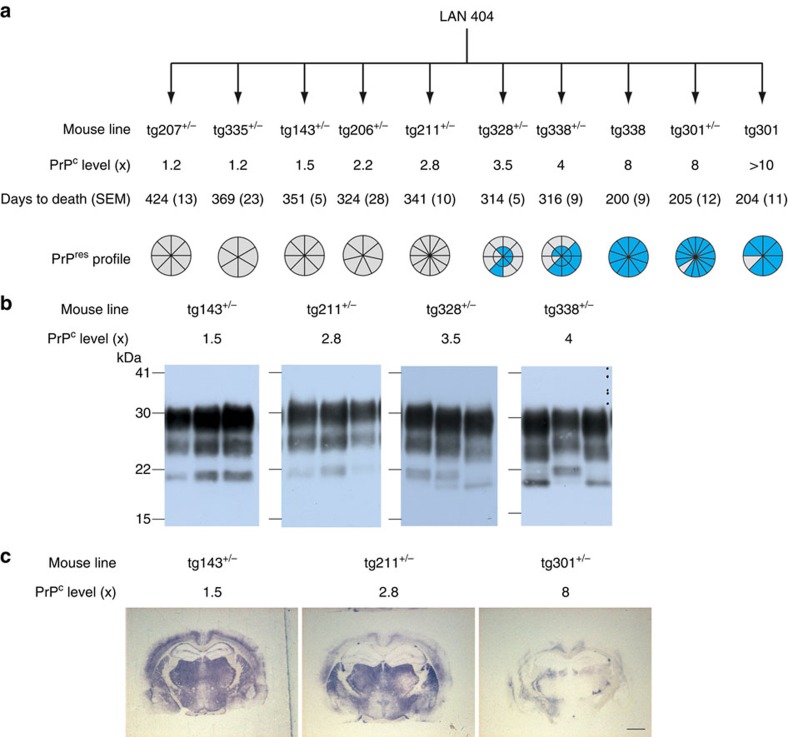
Survival time and brains PrP^res^ signatures in mice expressing PrP^C^ at varying levels on infection with LAN404 isolate. (**a**) Transmission of LAN404 to transgenic mouse lines expressing the VRQ allele of ovine PrP^C^ at varying levels (expressed as *x*-fold that in sheep brain). Segmented circles are used to indicate the proportion of mice with 19K (blue) or 21K PrP^res^ signature (grey) in their brains. A double circle is used to indicate the proportion of mice with mixed signature. (**b**) Representative western blots showing PrP^res^ glycopattern in the different mouse lines (the respective PrP^C^ expression levels are indicated). Mixed 21K and 19K signatures are seen in intermediate expresser mice (right two panels). (**c**) Histoblots of representative sections at the level of the hippocampus. The PrP^res^ deposition patterns in high and low (left two panels) expresser mice are distinct. Scale bar, 1 mm.

We next wondered whether or not the same phenomena would occur on transmission to an enlarged panel of lines all expressing the VRQ allotype, though from various transgene constructs (*tg1*, *tg2* and *tg3*), and at variable levels^36^. On including the *tg301*^+/−^ and *tg338* mice, the panel was comprised of 8 independent lines and 10 different genotypes, and the PrP^C^ expression level ranged from ∼1.2-fold to ≥10-fold that in sheep brain (Table 1). The results obtained after primary transmission of LAN404 are summarized in Fig. 3a. All the lines challenged intracerebrally with LAN404 developed clinical disease at 100% attack rate. There was an inverse correlation between the mean ID and PrP^C^ expression levels, as previously found on transmission of a *fast* field scrapie isolate to this same mouse panel^36^.

**Figure 4:**
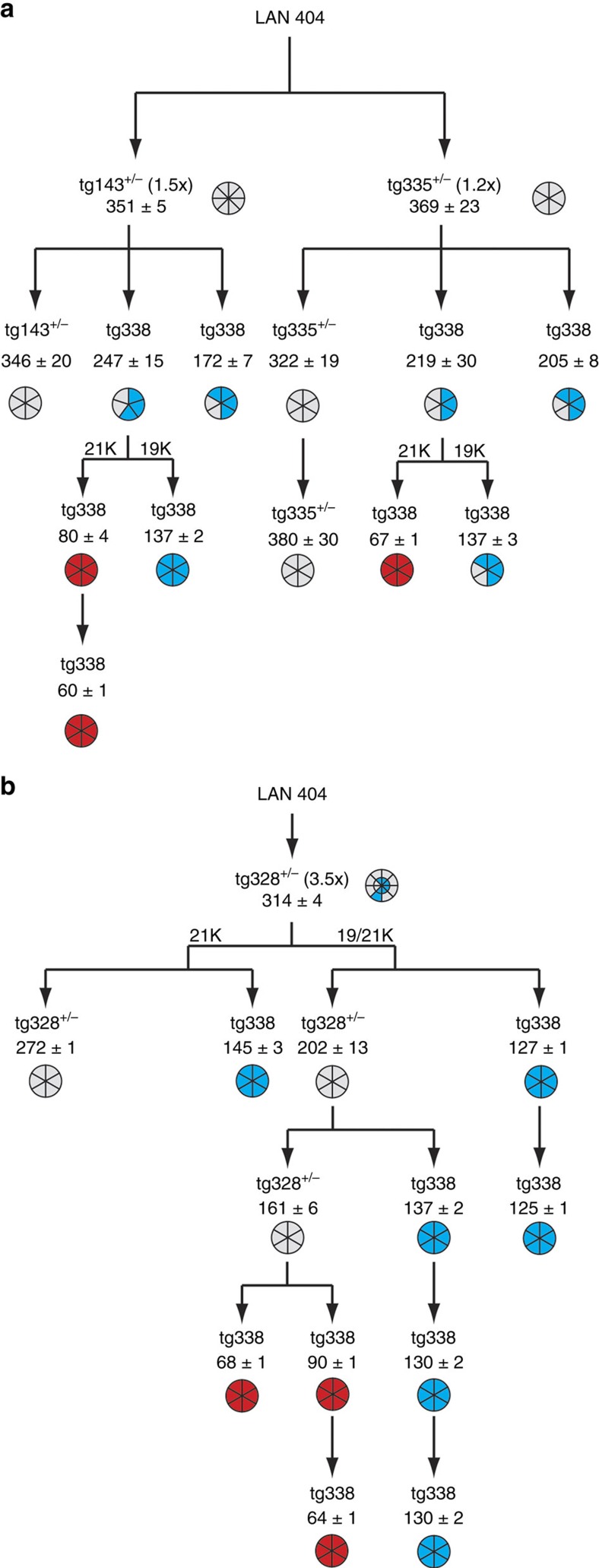
Serial transmission of LAN404 isolate to mice expressing ovine PrP^C^ at low and intermediate levels. (**a**) Serial transmission to low expresser mice and subpassage to high expresser mice (*tg338*). A phenotype associating 21K PrP^res^ and ID >300 days, designated *LA21K* (see main text), is maintained along subpassage on low expresser mice. (**b**) Serial transmission to mice expressing PrP^C^ at intermediate levels. Segmented circles are used to indicate the proportion of mice with *LA19K* (blue), *LA21K* (grey) or 21K *fast* (red) PrP^res^ profiles in their brains. A double circle is used to indicate the proportion of mice with mixed signature. Transmission of 21K PrP^res^ prions propagated on intermediate expresser mice (*tg328*^+/−^) to high expresser mice (*tg338*) enables to rescue both 19K and 21K *fast* phenotypes.

Most strikingly, the brain PrP^res^ profiles in the diseased mice differed among the lines and such a variation appeared to be primarily linked to the PrP expression level. Unlike that observed in overexpresser mice (≥8-fold), the 19K PrP^res^ signature was detected in none of the mice with an expression level below 3.5-fold (five independent lines, three different constructs); all these mice exhibited a 21K type signature in their brain, reminiscent of LAN404 and more enriched in unglycosylated species than *LA21K* *fast* in *tg338* mice (Fig. 1c). At intermediate expression levels (3.5- to 4-fold), mixed 19K and 21K PrP^res^ profiles were found in a notable proportion of mice (Fig. 3). This PrP^res^ signature discrepancy was observed in mouse lines harbouring the same (*tg3*) construct: 21K in *tg335*^+/−^ low expresser mice, 19K in nearly all *tg338* and *tg301*^+/−^ high expresser mice, and a combination of both in *tg328*^+/−^ and *tg338*^+/−^, intermediate expresser mice. Therefore, the transgene structure, and thus its expression pattern including in peripheral tissues such as spleen, played apparently no crucial, intrinsic role in this phenomenon. We then asked whether and how the phenotypes observed on low expresser mouse lines would evolve on secondary transmission. We performed transmissions from LAN404 primary inoculated, low expresser—*tg335*^+/−^ and *tg143*^+/−^—toward mice of the same line, and in parallel to *tg338* mice to see how low expresser mice-passaged agent would behave compared with LAN404 isolate (Fig. 4a). Remarkably, no abrupt shortening of the mean ID occurred when LAN404 was subpassaged on low expresser mice exclusively, and the PrP^res^ profile remained of 21K type uniformly (up to three passages on *tg335*^+/−^). On passage to *tg338* mice, however, the transmission pattern resembled that seen on LAN404 primary inoculated *tg338* and *tg301*^+/−^ mice (Fig. 1), that is, the 19K PrP^res^ profile prevailed (∼2/3 of the mice), and one additional passage led to the reappearance of either *LA19K* or *LA21K fast* uniform phenotypes, depending on whether brain tissue with 19K or 21K PrP^res^ was inoculated (Fig. 4a). Altogether, these results led us to conclude that: (i) mice expressing PrP at a sheep brain-like level do propagate preferentially an agent thereafter designated *LA21K*, which exhibits features of the original isolate; (ii) the *LA19K* agent is also able to replicate on low PrP^C^ level mice, however as a ‘hidden' component; (iii) *LA21K fast* agent can be re-isolated via transmission from low to high expresser mice.

We also performed serial transmissions within and from ‘intermediate' expresser mice. The resulting picture was overall consistent with the preceding results. Briefly, subpassages from primary inoculated *tg328*^+/−^ mice produced either 21K or19K type signatures, depending on whether the secondary inoculated mice were *tg328*^+/−^ or *tg338*, respectively. Even after two consecutive subpassages on *tg328*^+/−^ mice *LA19K* prions could be rescued and stabilized by using *tg338* mice for further passage (Fig. 4b). Essentially similar results were obtained on serial transmissions involving *tg338*^+/−^ mice (4-fold), which again behaved differently from their homozygous counterparts (Supplementary Fig. 2). These experiments showed that middle expresser mice allow silent propagation of *LA19K* agent for at least two subpassages.

### Three distinct strains can propagate on low expresser mice

**Figure 5:**
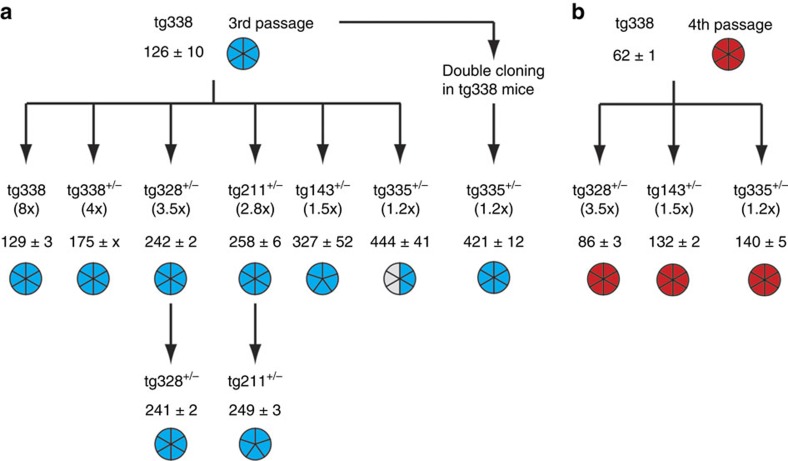
Transmission of *LA19K* and *LA21K fast* prions to a panel of mice expressing PrP^C^ at varying levels. *tg338*-passaged *LA19K* (uncloned or cloned, **a**) and *tg338*-passaged *LA21K fast* (**b**) were further transmitted to lines expressing PrP^C^ at variable levels, as indicated. Segmented circles: as in Figs 3 and 4. Both *LA19K* and *LA21K* fast prions can be faithfully propagated in low expresser mice.

As shown above, *LA19K* component lacked a selective advantage in mouse lines expressing PrP^C^ at levels below 4-fold. However, the question arose of whether such mice would allow a steady propagation of this agent first amplified on overexpresser mice. Brain material from three serial passages of LAN404 isolate on *tg338* mice was inoculated to relevant mouse lines (Fig. 5a). Remarkably, the PrP^res^ profiles were uniformly of 19K type in all but the lowest expresser tg335^+/−^ line. The mean ID tended to be briefer compared with unpassaged LAN404 inoculum, this difference increasing with the PrP^C^ expression level (Supplementary Table 1). Such results indicated that, while PrP^C^ overexpression is not a prerequisite for sustained replication of *LA19K* agent, the ID increases dramatically relative to 21K agent when PrP^C^ expression approaches a physiological level. The observed ‘resurgence' of 21K PrP^res^ signature in a proportion of *tg335*^+/−^ mice was likely due to residual 21K agent in *LA19K*-enriched inoculum. Indeed, when *LA19K* was biologically cloned on *tg338* mice before inoculation to *tg335*^+/−^ mice, the PrP^res^ profiles were of 19K type exclusively (Fig. 5a).

**Figure 6:**
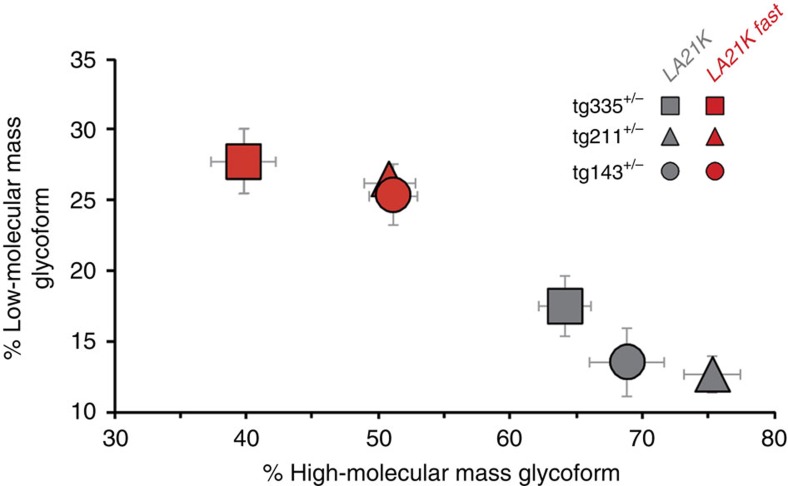
PrP^res^ glycoform patterns of *LA21K* versus *LA21K fast* prions in low expresser mice. Ratio of diglycosylated to monoglycosylated PrP^res^ species in the brains of *tg335*^+/−^ (square), *tg211*^+/−^ (triangle) and *tg143*^+/−^ (circle) mice following transmission of *LA21K fast* (red) or *LA21K* (grey) prions (data plotted as means±s.e.m.); *n*=5 mice analysed per prion strain and mouse line). *LA21K* and *LA21K fast* prions show distinct glycoform patterns, with little variation depending on the mouse line.

Since *LA21K* prions underwent no apparent, abrupt phenotypic change on propagation onto low expresser mice, it was feasible to compare their strain-specific traits with those of the *LA21K fast* variant emerging on mice expressing PrP^C^ at a ≥3-fold level. Inoculation to *tg143*^+/−^ and *tg335*^+/−^ mice of *LA21K fast* prions (four passages on *tg338* mice) produced mean IDs of 132±2 days and 140±5 days, respectively (Fig. 5b), thus markedly shorter than for LAN404 homogenate primary or secondary inoculated to the same lines (320–350 days; see Fig. 4). Further confirming that *LA21K* and *LA21K fast* are truly distinct strains, their glycoform-profiles were clearly differentiable in the brain of these mice, with *LA21K* being enriched in diglycosylated forms, as in the LAN404 primary isolate (Fig. 6).

**Figure 7:**
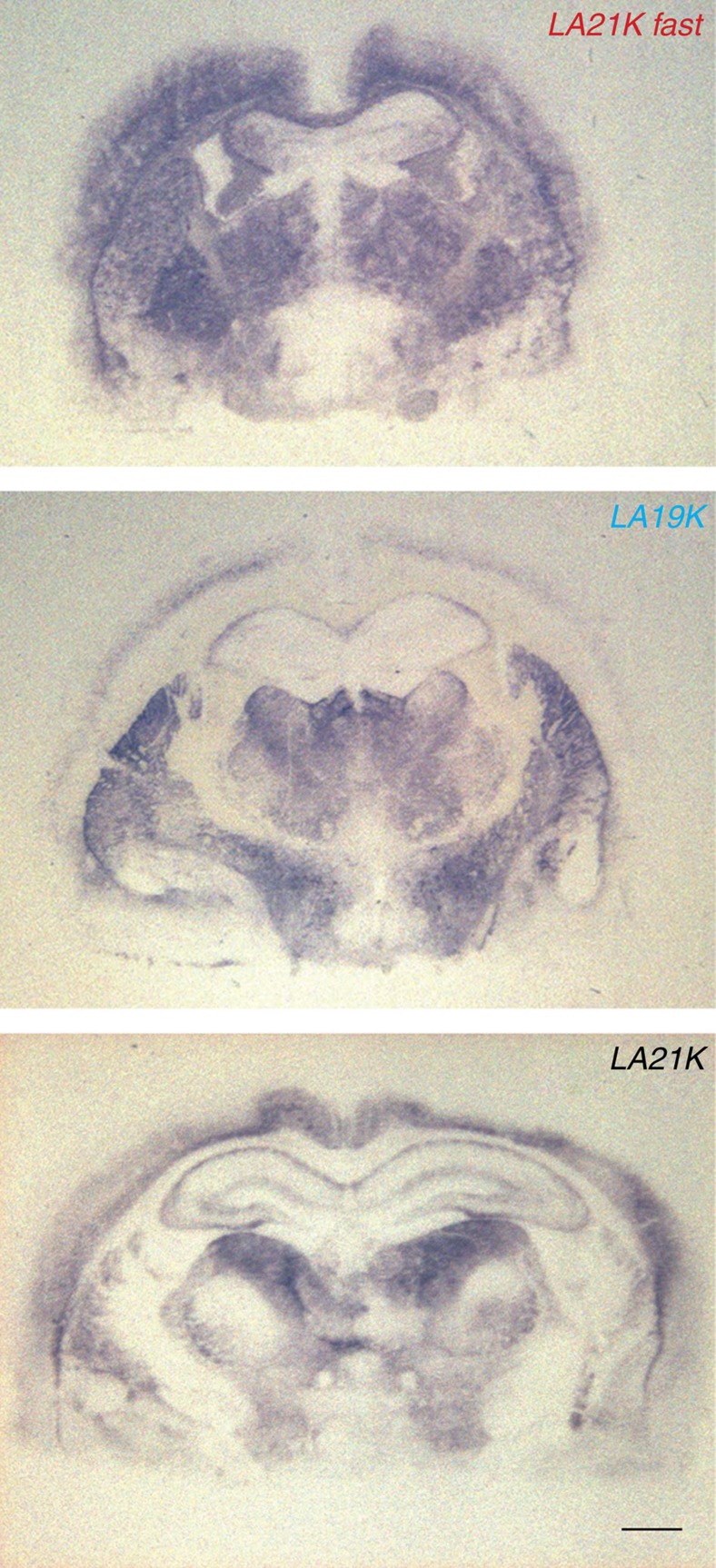
PrP^res^ deposition pattern in *tg335*^+/−^ mice inoculated with *LA21K fast* or *LA19K* or LA*21K* prions. Representative histoblots at the level of the hippocampus are shown. In *LA21K fast*-infected mice, there is a widespread and granular-like deposition of PrP^res^ in most brains areas, including the cortex and corpus callosum. The protein was not detected in the habenula. In *LA21K*-infected mice, PrP^res^ was not detected in the cortex and corpus callosum, while present in the habenula, thalamus and hypothalamus. In *LA19K*-infected mice, there is a preferential accumulation of fine PrP^res^ deposits in the thalamus, oriens layer of the hippocampus and cerebral cortex. Scale bar, 1 mm.

Inoculation to low expresser mice of *LA21K* fast and *LA19K* prions from early passages on high expresser mice produced results essentially similar to those described above (Supplementary Fig. 3). Finally, histoblotting analysis revealed distinct features for the three identified agents (Fig. 7). Altogether these results clearly show that not less than three distinct prion strains, all derived from the same isolate, could be propagated on low expresser mice.

## Discussion

In this study we provide experimental evidence that on homotypic transmission of a natural prion source to transgenic mice, different strains may be propagated depending on the PrP^C^ gene dosage in the recipient line. Indeed, we show that serial brain-to-brain transmission of a representative, sheep scrapie isolate to a panel of ten transgenic mouse lines that expressed the same ovine PrP^VRQ^ allotype at a varying level, 1- to 10-fold that in sheep brain, produced a pattern of unexpected complexity, involving no less than three distinct, *bona fide* prion strains. A mechanism of strain selection based on PrP^C^ expression level is proposed. Aside obvious implications regarding the characterization of natural prions by transmission to transgenic mice, our findings lead us to hypothesize that variation in the neuronal PrP^C^ expression level might contribute to the intriguing phenomenon of strain- specific, neuronal targeting exhibited by mammalian prions.

**Figure 8:**
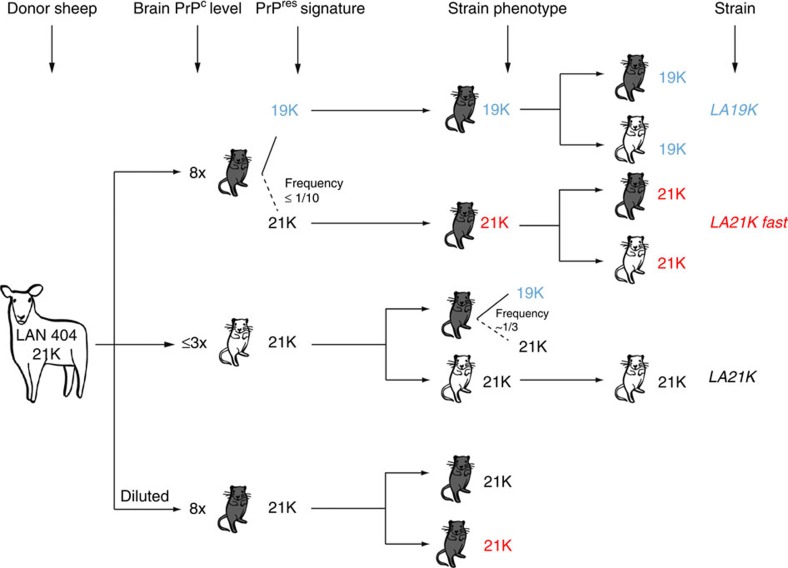
Transmission pattern of LAN404 scrapie isolate to transgenic mice expressing PrP^C^ at different levels. A schematic overview of the pattern observed upon homotypic transmission of this VRQ/VRQ sheep isolate to transgenic mice expressing same allele of ovine PRPC is shown. High expresser mice (8-fold level; expressed as *x*-fold that in sheep brain) and low expresser mice (≤3-fold level) are represented in grey and white, respectively. On high expresser lines, 19K prions (blue) subdominant in the donor sheep brain are preferentially propagated over 21K prions. 21K prions occasionally mutate to 21K *fast* prions (red). On lines expressing PrP^C^ at a physiological or ≤3-fold level, 21K prions (black) are preferentially propagated over 19K prions, as assumed to be the case in the donor sheep. Overall, transmission of LAN404 isolate led to the individualization of three strain components, *LA19K*, *LA21K* and *LA21K fast*, each of them being able to replicate—overtly or silently—on both high and low expresser lines. Overt propagation of *LA21K* prions in high expresser mice can be observed on inoculating diluted, primary inoculum so as to eliminate the subdominant *LA19K* component.

The somewhat intricate data presented here can be summarized as depicted in Fig. 8. Transmission of LAN404 isolate to high expresser mice led rapidly—as soon as at first passage—to the emergence of two obviously different strain components that proved to be fairly stable phenotypically on subsequent, iterative passage. The most parsimonious interpretation of the experiments subsequently carried out is that this dual evolution was determined by two distinctive events: (i) the preferential amplification of an agent designated *LA19K,* pre-existing in the brain tissue of the donor sheep as a subdominant component; (ii) the stochastic, *de novo* emergence in a minority of mice of an agent called *LA21K fast*, a ‘mutant' from the 21K strain component predominating in the donor sheep brain, which propagates even faster than *LA19K* agent. Strikingly, none of these agents emerged when the same isolate was serially passaged onto mouse lines expressing PrP^C^ at a sheep-like level or up 3-fold higher. Instead, such mice appeared to stably propagate an agent termed *LA21K*, with molecular properties of the original isolate, and from which it was possible to re-isolate the *LA19K* and *LA21K fast* components by transmission to high expresser mice. Nevertheless, transmission of *LA19K* and *LA21K fast* propagated on high expresser mice to low expresser ones resulted in faithful and steady propagation of both these agents.

The abrupt *LA21K* to *LA19K* strain shift following transmission to mice expressing PrP^C^ at supraphysiological levels implies that an authentic adaptation process had been taking place, mimicking what can occur after across species transmission^24^,^25^,^26^,^27^,^28^,^39^. In the present case, however, neither a divergence between the coding sequences of donor and host PrP^C^, nor an effect of the transgene organization could be incriminated. Consequently, the transgene dosage itself has to be considered as the key determinant. How could the availability in PrP^C^ substrate at a cell or tissue level variably affect prion propagation efficiency in a strain-dependent manner? Overexpression might favour one strain over another by causing extra- localization of PrP^C^ in specific brain region(s). This is unlikely since a BAC construct, known to lead to an integration-site independent and copy-number related level of expression^36^,^37^, was used in both high and low expresser *tg3* lines. Moreover, histoblotting analyses of uninfected mice harbouring the *tg3* construct (Supplementary Fig. 4) revealed a widespread distribution of PrP^C^ in the brain whatever the expression level, without obvious qualitative differences, notably in areas of preferential PrP^Sc^ accumulation for either one or the strains. Hence the ‘ectopic' explanation, that is, a competitive advantage in specific neural cell populations to which overexpression would give access, seems unlikely. Saturation of a binding partner is an alternative possibility that cannot be ruled out. According to the literature, a number of cellular factors can promote the replication efficiency to a variable extent depending on the strain^40^,^41^,^42^. Assuming that such a factor is both physically interacting with PrP^C^ and in limiting concentration, its exhaustion in overexpressing mice would afford a competitive advantage to the strain that is less dependent on it. We do favour, however, another mechanistic explanation involving a kinetic competition during prion fibres formation. How can a strain A predominate over a strain B only beyond a certain threshold of PrP^C^ concentration, without having an all-or-none phenotype? This can be mathematically modelled assuming that: (i) during the templating process, oligomeric PrP^C^ is incorporated according to a concerted process (discussed in ref. 42); (ii) the number of protomers *n* within the oligomeric subunits is a strain feature, with *nA*>*nB* (Supplementary Fig. 5 and Supplementary Note 1).

Unlike *LA19K*, *LA21K fast* appears to occasionally emerge as a mutant of the parental strain *LA21K* during the propagation in mice. The tendency of slow replicating strains to generate a faster mutant is documented for mammalian as well as yeast prions^25^,^32^,^43^. Why such a mutational event would be enhanced in overexpresser mice is uncertain, a possible explanation being that the shift toward the *LA21K fast* prion folding pathway involves an interaction with PrP^C^ molecules in a rare or transient conformational state.

To be underlined, the complex transmission pattern seen with LAN404 isolate is not a unique feature. On the contrary, the 19K/21K phenotype divergence on high/low expresser mice was observed with 18 out of 20 geographically unrelated isolates examined purposely. Moreover, LAN404 is the strain prototype of the group found to be the most abundant among five major groups delineated following transmission of a large panel of natural sheep typical scrapie isolates^9^). The nearly constant co- propagation of *LA21K* and *LA19K* agents thus suggests an ontogenic link, rather than a simple strain mixing^20^. No *LA21K* prion was found to emerge on serial propagation of biologically cloned *LA19K*. Conversely, *LA21K* could be the parent strain, with an inherent propensity to generate 19K prions, yet as a silent component in sheep brain. In contrast, the *LA21K* mutation into *LA21K fast* variant would have to be a rare event in natural conditions—at least on VRQ/VRQ sheep. In any case, it is of interest that the combined propagation of a single isolate on low and high expresser mice recapitulated part of the natural scrapie strains variation as documented by transmission to high expresser mice^9^. In particular, *LA19K* and the reference scrapie strain CH1641 exhibit identical features^44^. Thus panels of transgenic mice harbouring various PrP^C^ levels might represent a relevant tool in an attempt to learn more about the determinism underlying the diversity and evolution of natural prions. Incidentally, there is frequent co-occurrence of 21K/19K PrP^res^ signatures in the brain of human individuals affected by sporadic CJD whose strainness is still debated^18^,^45^,^46^. Our findings might contribute to clarify this issue. A further, methodological implication of our findings is that subpassaging of natural isolates using brain pools of infected mice—not an unusual practice in the field—should be avoided as it can produce truncated or misleading information.

The molecular basis of the strain-specific targeting of brain regions by prions remains unexplained to date. A commonly, long-invoked hypothesis, that is, the selective permissiveness of neural cell subpopulations, has been recently supported by elegant studies showing that the spectrum of infectible cell lines and subclones encoding the same PrP^C^ allele can markedly differ from one strain to another^47^. Various, still conjectural mechanisms have been advanced to account for such a strain discriminative, cell autonomous permissiveness, involving: (i) the array of available convertible PrP^C^ isoforms notably in terms of glycoform ratio^48^,^49^,^50^; (ii) the availability of co-factors influencing the replication efficiency^40^,^41^,^42^,^51^; (iii) the rate of PrP^Sc^ processing and clearance^52^,^53^; (iv) the polymer fragmenting activity resulting in new seeds^54^,^55^. Our findings lead us to conjecture that the steady state level of PrP^C^ might contribute to the differential permissiveness of specific neural subpopulations to prion (sub)strains, possibly on a replication dynamics basis. Indeed, the expression of PrP^C^ actually varies depending on the brain structure^48^,^56^,^57^,^58^,^59^. A marked disparity in the PrP^C^ steady state level (up to ≥10-fold) exists among neuronal subpopulations, the primary cause being seemingly the rate of degradation rather than the rate of synthesis^57^. As a very preliminary step to test the pathophysiological relevance of this novel notion, we examined the regional distribution of PrP^res^ in diseased mice infected with *LA19K* prions (Supplementary Fig. 6). Although PrP^res^ in the whole brain was 19K type, essentially 21K PrP^res^ was found to accumulate in the brainstem, where PrP^C^ is expressed at lower levels^48^. Moreover, our ongoing studies suggest that the PrP^C^ expression level in extraneural compared with nervous tissues might contribute to the selective neurotropism manifested by certain prions.

## Methods

### Ethics

Animal experiments were conducted in strict accordance with ECC and EU directives 86/009 and 2010/63 and were subsequently approved by the local ethics committee of the author's institution (Comethea; permit number 12/034).

### Transgenic mouse lines

The constructs, the mouse lines and their respective ovine PrP^C^ expression level (VRQ allele) have been described^36^,^60^,^61^, and are listed in Table 1. Female, 6–8 week-old individuals were used were used for the prion transmission experiments.

### Prion transmission

To avoid any cross-contamination, a strict protocol based on the use of disposable equipment and preparation of all inocula in a class II microbiological cabinet was followed. Brain material (brainstem) from sheep terminally affected with natural scrapie^36^ sources was used as prion sources. The tissue extract was prepared as 10% w/v homogenate in 5% w/v glucose with a Hybaid or Precellys rybolyzer (Ozyme, Montigny-le-Bretonneux, France) for inoculation to ovine PrP transgenic mice. Twenty microliters were inoculated intracerebrally in the right hemisphere to groups of individually identified mice, at the level of the parietal cortex. For subsequent passage, mouse brains were collected with sterile, disposable tools, homogenized at 20% w/v in 5% glucose and reinoculated intracerebrally at 10% w/v. For endpoint titration, starting from 10% w/v brain homogenate (undiluted material), serial 10-fold dilutions of brain homogenates were prepared extemporaneously in 5% w/v glucose containing 5% w/v bovine serum albumin. Twenty microliters of each dilution were inoculated into recipient mice by intracerebral route. Animals were supervised daily for the prion disease development. Animals at terminal stage of disease were euthanized. Their brains were analysed for proteinase K-resistant PrP^Sc^ (PrP^res^) content.

### Western blot

Brain homogenates (typically 200 μl of 10% brain homogenate) were digested for 1 h with PK (final concentration 10 μġml) at 37 °C. The reaction was stopped with 4 mM PMSF. After addition of 10% sarcosyl and 10 mM Tris–HCl (pH 7.4), samples were incubated for 15 min at room temperature. They were centrifuged at 245,000*g* for 45 min at 20 °C on 10% sucrose cushions. Pelleted material was resuspended in sample buffer, resolved by 16% Tris-tricine gels, electrotransferred onto nitrocellulose membranes, and probed with anti-PrP monoclonal antibodies with epitope within PrP globular domain: 2D6 (human PrP epitope 140–160 (ref. 62), Sha31 (human PrP epitope 145–152 (ref. 63)). Immunoreactivity was visualized by chemiluminescence. Determination of PrP^res^ glycoform ratios was performed with the GeneTools software after acquisition of the signals with a GeneGnome digital imager.

### Histoblots

Brains were rapidly removed from euthanized mice and frozen on dry ice. Cryosections were cut at 8–10 μm, transferred onto Superfrost slides and kept at −20 °C until use. Histoblot analyses were performed as described^30^, using the 12F10 anti-PrP antibody^64^ (human PrP epitope 142–160). Analysis was performed with a digital camera (Coolsnap, Photometrics) mounted on a binocular glass (SZX12, Olympus). The sections presented are representative of the analysis of three brains samples. The protocol was similar to study PrP^C^ distribution, except that the proteinase K treatment step was omitted^65^. The scale bar shown in Fig. 1 applies to all histoblots.

### Data availability

The authors declare that all data supporting the findings of this study are available within the paper and its Supplementary Information files, or available from the authors upon reasonable request.

## Additional information

**How to cite this article:** Le Dur, A. *et al*. Divergent prion strain evolution driven by PrP^C^ expression level in transgenic mice. *Nat. Commun.* **8,** 14170 doi: 10.1038/ncomms14170 (2017).

**Publisher's note**: Springer Nature remains neutral with regard to jurisdictional claims in published maps and institutional affiliations.

## Supplementary Material

Supplementary Information Supplementary Figures, Supplementary Tables, Supplementary Notes and Supplementary References

## Figures and Tables

**Table 1:** Characteristics of the sheep PrP^VRQ^ transgenic mouse lines used.

Mouse line*	**Transgene construct**†	**Genotype**	**Brain PrP**^**C**^ **expression**‡
*tg301*	bac*Prnp*	+/+	>10
		+/−	8
*tg338*	bac*Prnp*	+/+	8
		+/−	4
*tg328*	bac*Prnp*	+/−	3.5
*tg211*	cmv/phg*Prnp*	+/−	2.8
*tg206*	cmv/phg*Prnp*	+/−	2.2
*tg143*	phg*Prnp*	+/−	1.5
*tg335*	bac*Prnp*	+/−	1.2
*tg207*	cmv/phg*Prnp*	+/−	1.2

^*^All these mouse lines have already been described^30^,^36^. Two of them were used at either the heterozygous or homozygous state (quoted *tg338* and *tg301* in the main text).

^†^Mouse *Prnp*^0/0^ background (*Zürich I*).

^‡^Expressed as *x*-fold that in sheep brain. The respective brain PrP^C^ levels were reassessed, and found identical to that previously published, except in the case of *tg211*^+/−^ mice (2.8- instead of 1.9-fold).
