# Supplementary material for: Divergent prion strain evolution driven by PrPC expression level in transgenic mice
Source: Nat Commun. 2017 Jan 23;8:14170. doi: 10.1038/ncomms14170 (PMC5264111; doi:10.1038/ncomms14170)
Supplement: Supplementary Information — Supplementary Figures, Supplementary Tables, Supplementary Notes and Supplementary References [file ncomms14170-s1.pdf]

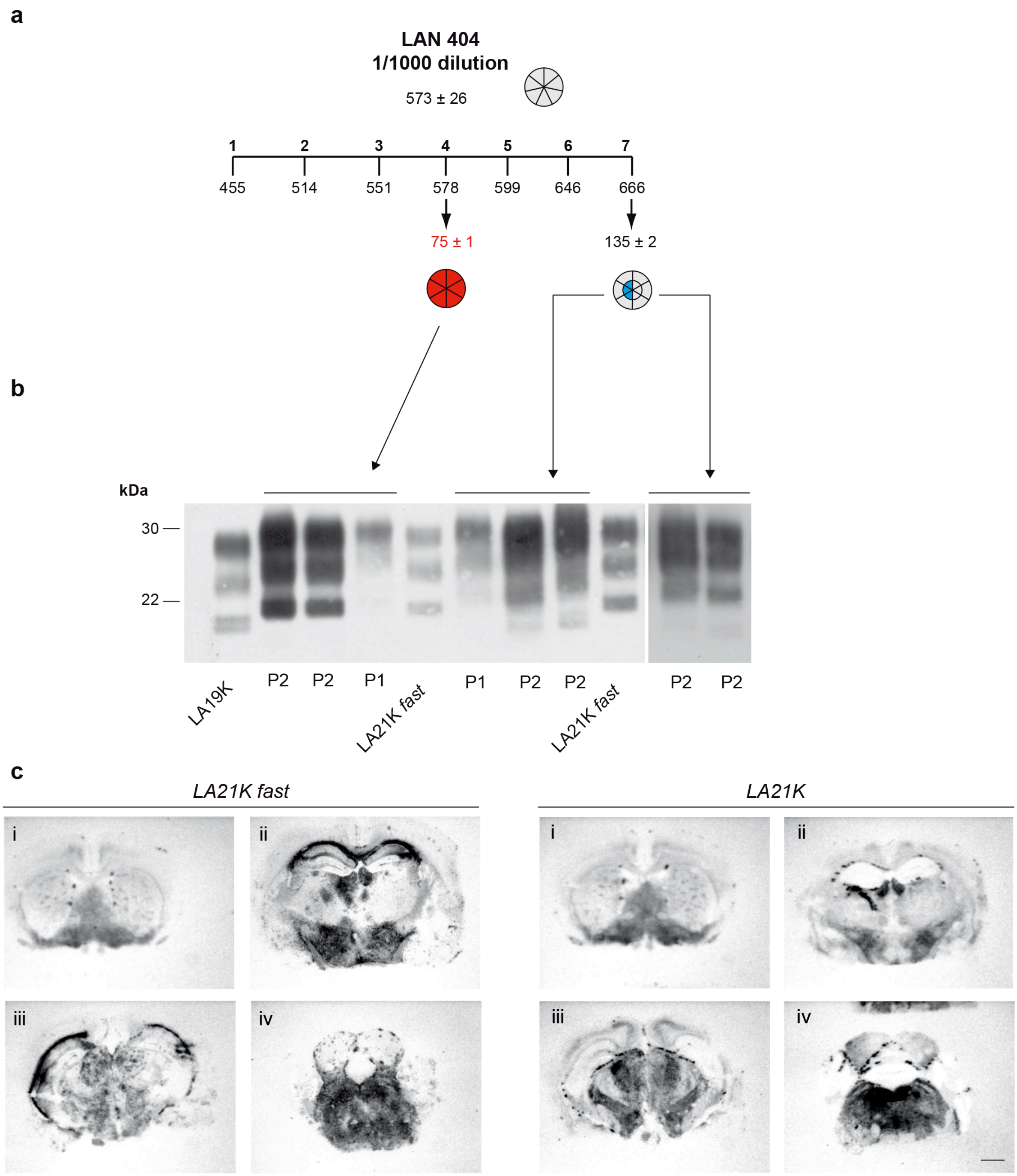

Supplementary Figure 1. Transmission of diluted LAN404 sheep scrapie isolate to transgenic mice overexpressing ovine PrP.

(**a**) Primary transmission of 1:1000 diluted LAN404 sheep scrapie isolate and subpassage to *tg338* mice (6 mice per brain inoculated). On primary transmission, all the mice showed a 21K PrP^{res} signature in their brain. On secondary transmission, made with undiluted brain homogenate from mouse no. 4 and 7, divergent phenotypes were observed: either a short ID

typical of *LA21K fast* prions (red), or a longer ID together with 21K (grey) and mixed 21K/19K (grey + blue) PrP^{res} profiles. (**b**) PrP^{res} electrophoretic patterns at primary passage (P1) and secondary passage (P2). The 21K PrP^{res} signatures (fragment size, glycoform ratio) are similar between P1 and P2 for mouse no.7, whereas for mouse no.4 the brains exhibit a *21K fast*-like signature at P2. Electrophoretic patterns of *LA21K fast* and *LA19K* prions serially passaged in *tg338* mice are shown as controls. (**c**) Representative histoblots (2nd passage) of antero-posterior coronal brain sections at the level of the septum (i), hippocampus (ii), midbrain (iii) and brainstem (iv). The pattern observed at P2 for mouse no.4 is specific of *LA21K fast*, with prominent PrP^{res} deposition in the corpus callosum, lateral hypothalamic nuclei and raphe nuclei of the brain stem. The pattern observed at P2 for mouse no.7 is different, with plaque-like PrP^{res} deposits scattered in the corpus callosum, the dorsal thalamus, the optic tract, the periaqueductal area, the tegmental nuclei and the cerebellum. Scale bar = 1 mm.

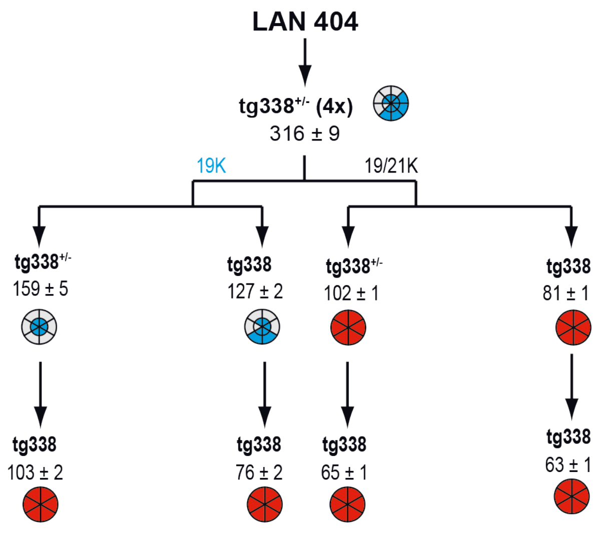

Supplementary Figure 2. Serial transmission of LAN404 scrapie isolate to mice expressing ovine PrP at intermediate levels.

Transmission to middle expresser *tg338*$^{+/-}$ mice and subpassage to the same line or to high expresser mice (*tg338*). Segmented circles are used to indicate the proportion of mice with *LA19K* (blue), *LA21K* (grey) or *21K fast* (red) PrP^{res} profiles in their brains. A double circle is used to indicate the proportion of mice with mixed signature. Middle expresser mice allow silent propagation of *LA19K* agent for at least two subpassages.

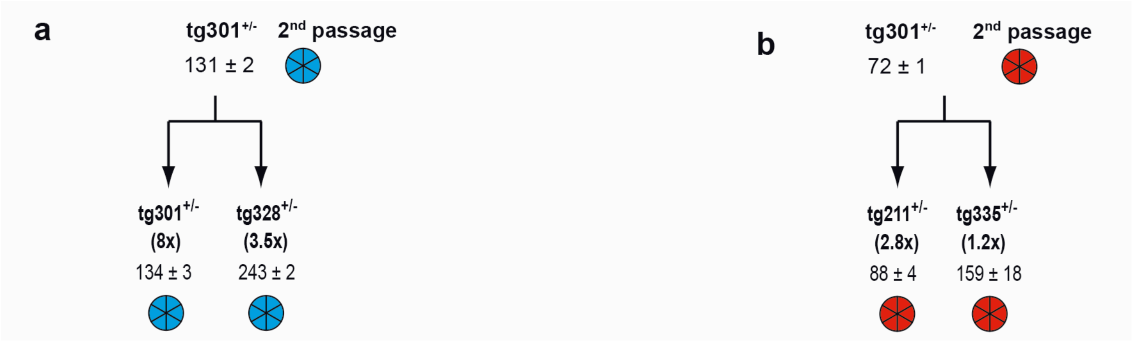

Supplementary Figure 3. Transmission of *LA19K* and *LA21K fast* prions to mice expressing ovine PrP at middle or low levels.

LA19K (blue) and *LA21K fast* (red) prions at the second passage on *tg301*$^{+/-}$- (see **Fig. 1a**) were further transmitted to lines expressing PrP^{C} at lower levels, as indicated. Segmented circles are used to indicate the proportion of mice with *LA19K* (blue) or *LA21K fast* (red) PrP^{res} profiles in their brains. Both *LA19K* and *LA21K* fast prions can be faithfully propagated in low or middle expresser mice.

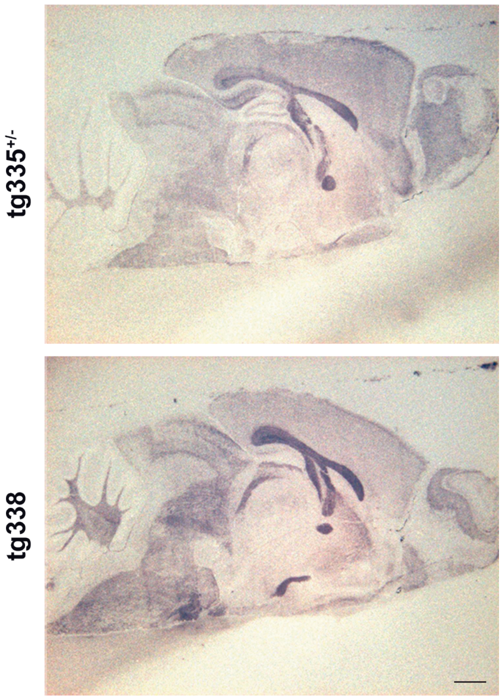

Supplementary Figure 4. PrP^{C} expression pattern in low and high expresser PrP mice.

Histoblots of representative sagittal sections from *tg335*$^{+/-}$ mice and *tg338* mice.

Scale bar = 1 mm.

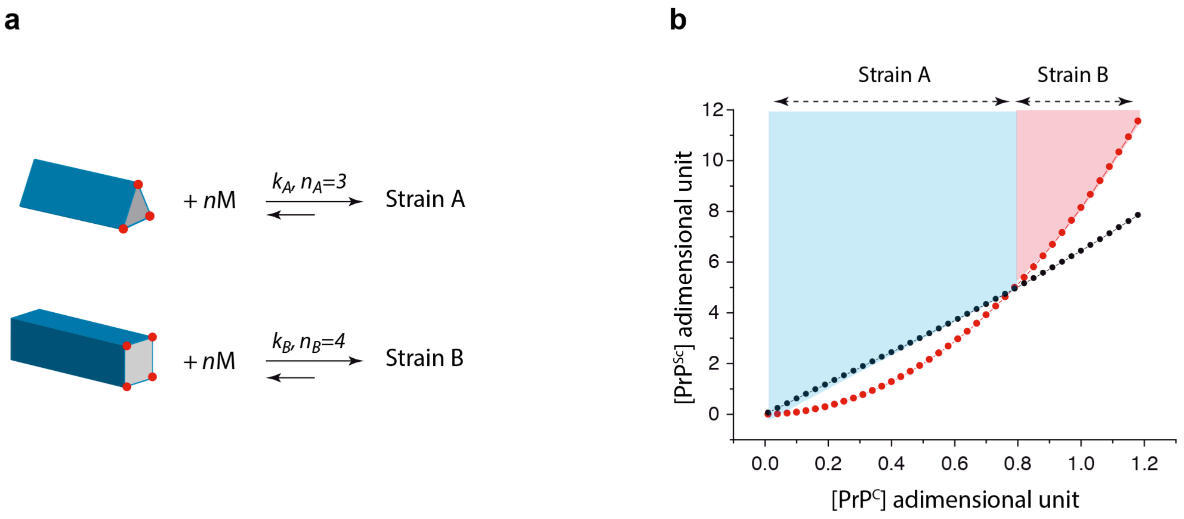

Supplementary Figure 5. Modelling strain-dependent polymerization rate as a function of PrP^{C} levels.

(**a**) Kinetic scheme of the conversion process of substrains A and B with two different kinetic order (n_A= 3 and n_B= 4). k_A and k_B correspond to conversion kinetics rate constants. (**b**) The simulation of PrP^{Sc} accumulation for substrains A and B as a function of PrP^{C} concentration reveals that below certain amount of PrP^{C}, strain A is the dominant population whereas at high PrP^{C} concentration strain B is the dominant population. For more details see **Supplementary Note 1**.

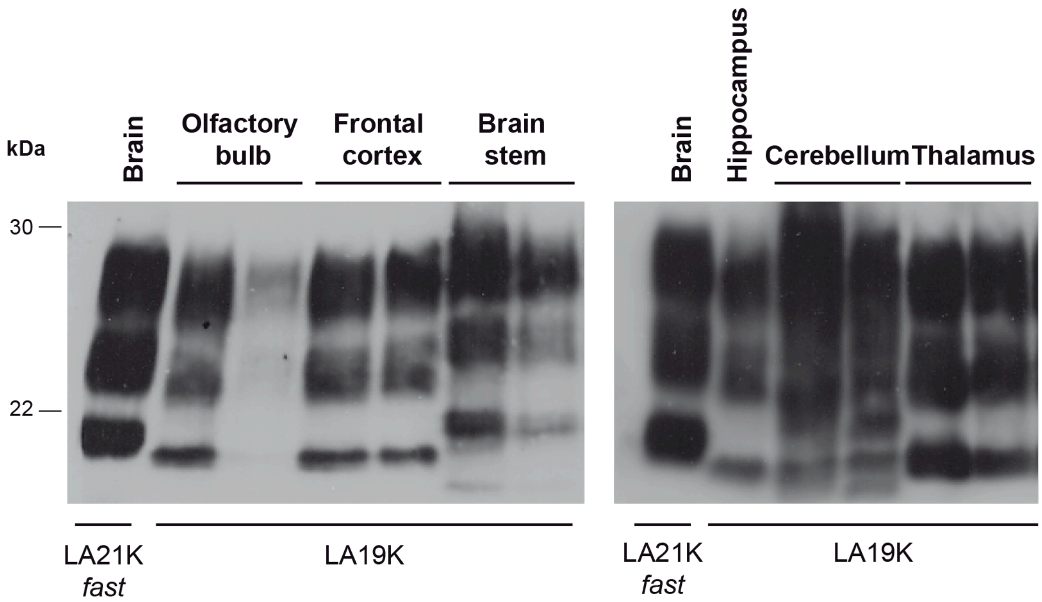

Supplementary Figure 6. PrP^{res} glycopattern in different mouse brain regions of *tg338* mice inoculated with an uncloned LA19K prion source (3rd passage in *tg338* mice). The brain stem of the infected mice was enriched in PrP^{res} species with a 21K signature, and prominent proportion of diglycosylated species as compared to *LA21K fast* (shown as control). A mixed 21K/19K profile was observed in the cerebellum.

Supplementary Table 1: Duration of disease in mice expressing PrP^C at variable levels, inoculated with LAN404 sheep brain or *tg338*-derived LAN404 prions (2nd passage, 19K PrP^{Sc} signature).

Transgenic mouse line	PrP^C level*	Days to death		Difference
		tg338-LA19K	LAN404	
$tg338^{+/-}$	4	175	316	-141
$tg328^{+/-}$	3.5	242	314	-72
$tg211^{+/-}$	2.8	258	341	-83
$tg143^{+/-}$	1.5	327	351	-24
$tg335^{+/-}$	1.2	444	424	+20

*Expressed as x-fold that in sheep brain

Supplementary Note 1. Kinetic simulation of substrain selection as a function of PrP^C expression level.

In order to simulate the outcome of LAN404 isolate transmission to transgenic mice expressing PrP^C at different, levels, we assumed the following:

1- Two substrains A and B exist in the same isolate.

2- The kinetic order of the templating process for each substrain is different. The replication of substrain A occurs by the integration of three PrP^C molecules (n=3) when for substrain B, four molecules are required (n=4). This replication mode contrasts with the generic model proposed by Weissman and colleagues for Sup35, for which the templating occurs through the integration/conversion of one monomer[1]. Recent developments in our group strongly suggest that mammalian prion assemblies are constituted of oligomeric, elementary bricks. As an example, for 263K hamster prions, this elementary brick would correspond to a trimer (Igel-Egalon et al., to be published).

3- The conversion rate remains constant during the evolution of the pathology

4- The fragmentation rate is not considered in order to have a constant conversion process.

5- PrP^C expression level does not vary (overexpressing mice) or to a limited extent during the early phase of replication[2].

Then we defined C_i and $\bar{C}_i$ respectively as the concentration of substrain A and B. i defines the size of assemblies as was defined in the simulation as adimensional parameter.

Kinetics constants used for simulations are :

k1 = 100 $M^{-3}s^{-1}$	k2 = 0.16 s^{-1}	n=3
k3 = 1.05 $M^{-4}s^{-1}$	k4= 0.01 s^{-1}	p=4

Simulations were run using ODE 15s from MATLAB routine for a concentration range of 10^{-8} to 10^{-6} as µM. Results were reported as adimentional variable. Phase space were analysed using Jacobin and Rout-Hurwitze criteria using a homemade MATHLAB code.

$$C_i + nC_1 \xrightarrow{k_1} C_{i+n}$$

$$\tilde{C}_i + pC_1 \xrightarrow{k_3} \tilde{C}_{i+p}$$

$$C_{i+n} \xrightarrow{k_2} C_{i+n-1} + nC_1$$

$$\tilde{C}_{i+p} \xrightarrow{k_4} \tilde{C}_{i+p-1} + pC_1$$

$$\frac{dC_{i+n}}{dt} = k_1 C_i C_1^n - k_2 C_{i+n}$$

$$\frac{d\tilde{C}_{i+p}}{dt} = k_3 \tilde{C}_i C_1^p - k_4 \tilde{C}_{i+p}$$

$$\frac{dC_1}{dt} = k_2 C_{i+n} + k_4 \tilde{C}_{i+p} - (k_1 C_i C_1^n + k_3 \tilde{C}_i C_1^p) + U$$

References

1 Collins, S. R., Douglass, A., Vale, R. D. & Weissman, J. S. Mechanism of prion propagation: amyloid growth occurs by monomer addition. *PLoS Biol* **2**, e321, doi:10.1371/journal.pbio.0020321 (2004).

2 Mays, C. E. *et al.* Prion Infectivity Plateaus and Conversion to Symptomatic Disease Originate from Falling Precursor Levels and Increased Levels of Oligomeric PrPSc Species. *J Virol* **89**, 12418-12426, doi:10.1128/JVI.02142-15 (2015).
